# Application of photobiomodulation in post-stroke dysfunctions: a literature review of recent clinical studies

**DOI:** 10.3389/fneur.2026.1859831

**Published:** 2026-08-14

**Authors:** Chao Ren, Xiaohong Chi, Yuan Jiang

**Affiliations:** 1 Rehabilitation Medicine Center, The Traditional Chinese Medicine Hospital of Longquanyi, Chengdu, Sichuan, China; 2 Department of Rehabilitation Medicine, Clinical Medical College and The First Affiliated Hospital of Chengdu Medical College, Chengdu, Sichuan, China

**Keywords:** aphasia, cognitive impairment, photobiomodulation, spasticity, stroke

## Abstract

Stroke patients often experience varying degrees of dysfunctions after a stroke, including motor, cognitive, speech, and other functional impairments. These post-stroke dysfunctions seriously affect the daily life and quality of life of patients. For many years, researchers have devoted themselves to finding non-invasive treatment methods to improve these functional deficits and enhance patients’ quality of life. Photobiomodulation (PBM) is a non-invasive phototherapy, and can be an adjuvant therapy for various neurological disorders by promoting neurogenesis and eliciting anti-apoptotic, anti-inflammatory, and antioxidative responses. Existing animal experimental evidence indicates that PBM may offer potential benefits for stroke patients. However, robust clinical evidence remains limited. Recent clinical studies provided new data supporting the efficacy of PBM in post-stroke dysfunctions, including motor, cognitive, and speech impairments. These studies used transcranial PBM (tPBM) for cognitive impairment and aphasia, or peripheral PBM directly to paretic limbs to yield positive outcomes for limb spasticity and pain. This mini-review summarizes key findings from these studies and discusses the associated challenges and prospects of PBM.

## Introduction

Photobiomodulation (PBM), also known as low-level light therapy (LLLT), is a non-invasive phototherapy employing red or near-infrared light (600 to 1,100 nm) at relatively low power densities to treat disease (1). Initially, PBM was used as an adjuvant therapy for wound healing, pain relief, musculoskeletal disorders, and more. Subsequently, an increasing number of studies have confirmed that transcranial PBM (tPBM) has shown good efficacy in treating various neurological disorders such as stroke, Alzheimer’s disease (AD), Parkinson’s disease (PD), traumatic brain injury (TBI), epilepsy, and even depression (2–4). During tPBM, light penetrates the scalp, periosteum, skull, meninges, and dura to reach the brain’s cortical surface, thereby inducing neurobiological changes. Scholars believe that tPBM is a safe intervention with no observed deleterious effects on the brain structure and function, and can provide medical benefits for central nervous system diseases by promoting neurogenesis and eliciting anti-apoptotic, anti-inflammatory, and antioxidative responses (5). Stroke is a leading cause of disability and mortality in the global adult population. Most survivors experience varying degrees of motor, cognitive, speech, and other functional impairments, which seriously affect the quality of life. The observed improvement of motor and cognitive functions with PBM treatment in animal models has stimulated people’s enthusiasm for researching PBM treatment for stroke patients (1). However, the results of three well-known clinical trials from over a decade ago, the NeuroThera Effectiveness and Safety Trials (NESTs), failed to provide convincing clinical evidence. NEST-1, NEST-2, and NEST-3 used PBM within approximately 24 h of ischemic stroke, and only NEST-1 and NEST-2 showed some patient benefit (6). However, NEST-3 was terminated early due to a lack of efficacy (5, 7). Some scholars suggested that significant differences in skull thickness and light-penetration characteristics exist between humans and animals, which directly influence the translation of PBM benefits from animal models to clinical practice (8). Recent clinical studies indicate that PBM can ameliorate various post-stroke dysfunctions, such as motor, cognitive, and speech impairments, thereby improving patients’ quality of life. These findings provide new clinical evidence for PBM’s efficacy in stroke patients, warranting further consideration and discussion. This mini-review summarizes key findings from clinical research on PBM for post-stroke dysfunctions over the past decade and discusses the associated challenges and prospects.

## Mechanism of action and neurophysiological effects of PBM

**Figure 1:**
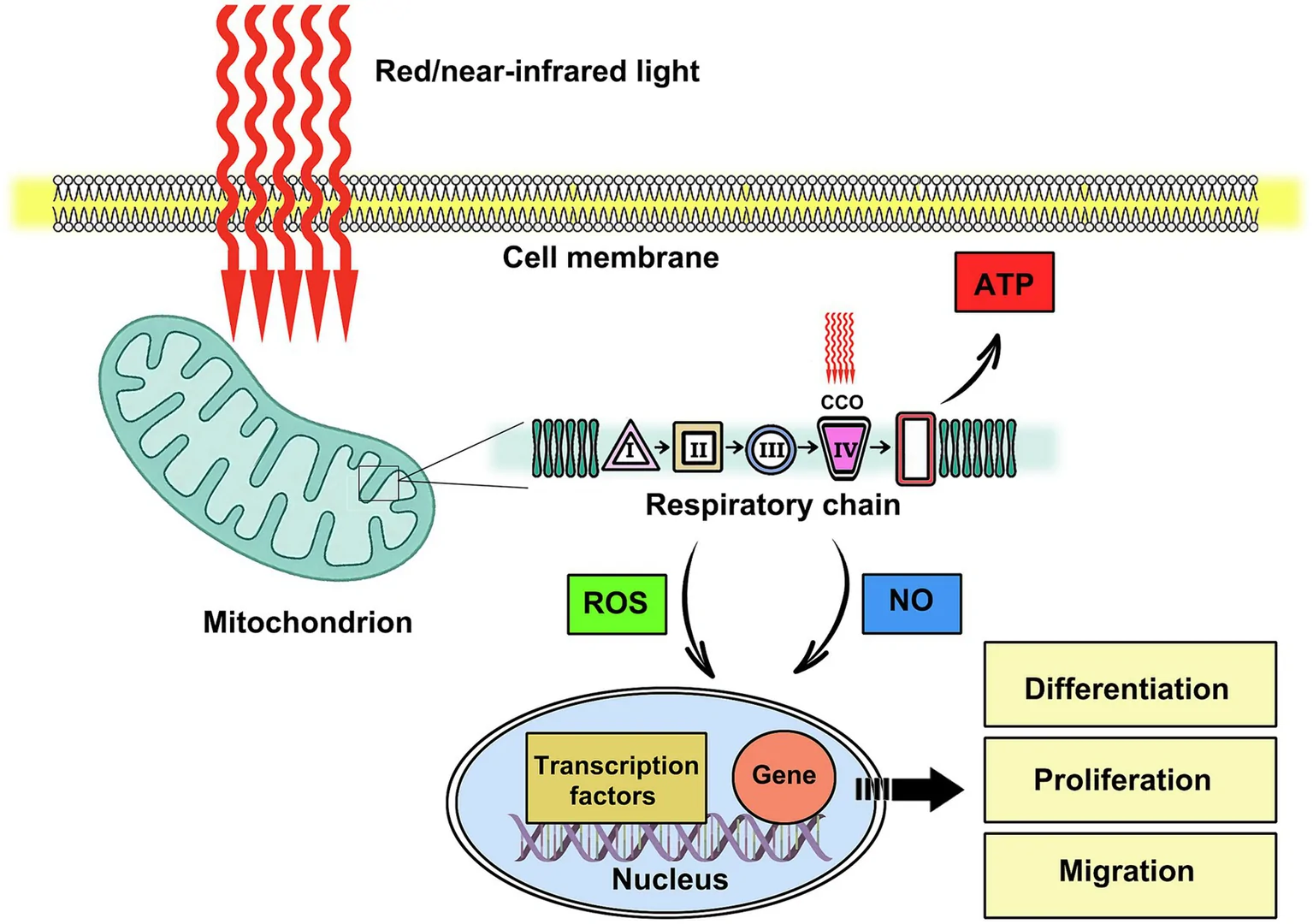
Schematic diagram of the mechanism of action of PBM.

Currently, it is widely believed that the mitochondrial effects of PBM are primarily mediated by photon absorption in cytochrome c oxidase (CCO), the main mitochondrial chromophore for red light (9). As a component of mitochondrial respiratory complex IV, CCO is responsible for transferring electrons from cytochrome c to molecular oxygen (O^2^). O^2^ is converted into water through glucose metabolism, generating ATP in the process. CCO enzyme activity can be impeded by nitric oxide (NO) in the presence of hypoxia or cellular damage, leading to disrupted electron transport efficiency, reduced mitochondrial membrane potential (MMP), and reduced ATP production (2). When CCO absorbs the photons from PBM within the red (600–700 nm) and near-infrared (760–940 nm) spectral ranges, inhibitory NO from CCO’s binuclear center (heme a3/CuB) can be photodissociated, leading to an increase in the MMP, consequently increasing the proton gradient and mitochondrial ATP production. This process triggers the release of second messengers, including NO, calcium ions (Ca2^+^), and reactive oxygen species (ROS), which activate signaling pathways such as nuclear factor-kappa B (NF-κB), p38 mitogen-activated protein kinases (MAPK), and protein kinase D2 (PRKD2), thereby regulating downstream cellular responses like differentiation, proliferation, and migration (10–12)(Figure 1). Additionally, PBM triggers a retrograde mitochondrial signaling that induces stimulated mitochondria to signal the cell nucleus to alter gene expression to augment mitochondrial function and biogenesis, ultimately triggering a cascade of intracellular changes, affecting ATP synthesis, pH, cAMP levels, and intracellular redox potential (13). Scholars also explored the non-mitochondrial effects of PBM, which involve signalling pathways that originate from photon absorption and protein conformational modulation at the cell membrane. For example, PBM could impact transient receptor potential (TRP) channels, which are pleiotropic cellular sensors to various external stimuli (e.g., light, pressure, and heat) and are involved in many cellular processes (14).

Within brain tissue, PBM boosts neuronal metabolic capacity and coordinates antioxidant, anti-inflammatory, and anti-apoptotic responses, ultimately promoting angiogenesis, neurogenesis, and synaptogenesis, which depend on its neurophysiological effects (15, 16). First, PBM generates a controlled amount of ROS, inhibits NF-κB and other signaling pathways, and ultimately alleviates neurological damage and facilitates the restoration of neural function by suppressing inflammation, reducing oxidative stress, and promoting neuronal regeneration (17). Second, PBM interacts with apoptosis signaling pathways to modulate the Bax/Bcl-2 ratio, reducing neuronal apoptosis and exhibiting neuroprotective effects. Third, PBM widens blood vessel diameter and enhances cerebral blood flow by elevating NO levels, while promoting angiogenesis by upregulating hypoxia-inducible factor 1α (HIF-1α) and vascular endothelial growth factor (VEGF), thereby improving tissue oxygenation and metabolic waste clearance (2). Fourth, PBM stimulates meningeal lymphatic vessels and enhances glymphatic drainage efficiency, thereby enhancing cerebral waste clearance and alleviating chronic inflammation in brain tissue.

## Application of PBM in post-stroke dysfunctions

Stroke patients often experience diverse dysfunctions due to different locations of lesions in the brain, including limb paralysis, aphasia, swallowing difficulties, cognitive impairments, and psychiatric diseases. These dysfunctions profoundly impact patients’ daily living ability and quality of life (18). Over the past decade, several clinical studies have reported that PBM can ameliorate post-stroke dysfunctions (Table 1). The paradigm of PBM includes transcranial and peripheral PBM in these studies.

**Table 1:** Literature examples of photobiomodulation clinical studies on post-stroke complications in the past 10 years.

Dysfunction	Light parameters	Irradiation site	Duration time	Assessment tools	Outcomes	Serious adverse events	References
Cognitive impairment	Equipment: laser Cluster; Wavelength: 660, 808, and 980 nm; Power density: 400.8 mW/cm^2^; Energy density: 24 J/cm^2^	Head with 15 irradiation regions	Each irradiation region for 60 s; Once a week, for 3 months.	Thermography; Mini-Mental State Examination (MMSE); Functional Independence Measure Scale™ (FIM)	Increased temperature in the irradiation regions; Increased cognitive function	Not mentioned	(21)
Cognitive impairment	Equipment: LEDs; Wavelength: 630 ± 15 nm; Power density: anterior helmet LEDs 20 mW/cm^2^, posterior helmet LEDs 40 mW/cm^2^, temporal helmet LEDs 5 mW/cm^2^, abdominal LEDs 5 mW/cm^2^; Energy density: anterior helmet LEDs 36 J/cm^2^, posterior helmet LEDs 72 J/cm^2^, temporal helmet LEDs 9 J/cm^2^, abdominal LEDs 9 J/cm^2^	Head and abdomen	Each irradiation region for 30 min; 5 times a week, for 3 months.	Mini-Mental State Examination (MMSE); Montreal Cognitive Assessment (MoCA); National Institutes of Health Stroke Scale (NIHSS); Blood and urine biomarkers: formaldehyde (FA), formaldehyde dehydrogenase (FDH), emicarbazide-sensitive amine oxidase (SSAO), cytochrome c (Cyt-c), hydrogen peroxide (H2O2), and coenzyme Q10 (CoQ10)	Improved cognitive function by modulating FA metabolism	No adverse events	(24)
Aphasia	Equipment: LED cluster; Wavelength: 633 nm (red LEDs), 870 nm (NIR LEDs); Power density: 22.2 mW/cm^2^; Energy density: 24 J/cm^2^	Protocol A: Bilateral (LH and RH) LED placements, and midline placements including both L and R supplementary motor areas (SMAs) at vertex; Protocol B: Only LH ipsilesional LED placements; Protocol C: Only LH, plus one midline cortical node of DMN (mPFC); Protocol D: Only LH, plus two midline cortical nodes of DMN (mPFC and precuneus)	Each protocol: 3 times a week, for 18 sessions total	Category naming; Picture naming; rs-fcMRI scans	Improved naming ability was present with optimal Protocol D	Not mentioned	(26)
Limb spasticity	Equipment: diode laser; Wavelength: 808 nm; Power density: 3.18 W/cm^2^; Energy density: 4.77 J/cm^2^	Rectus femoris and vastus muscles; 30 irradiation points for each muscle	Each irradiation point for 40 s	Isometric testing; Blood lactate level test	Increased peak torque; Decreased blood lactate level	Not mentioned	(28)
Limb spasticity	Equipment: diode laser; Wavelength: 808 nm; Power density: 3.18 W/cm^2^; Energy density: 127.39 J/cm^2^	Rectus femoris and vastus medialis muscles; 30 irradiation points for each muscle	Each irradiation point for 40 s	Visual analogue scale (VAS); Isometric testing; Surface electromyography (EMG)	Increased recruitment of muscle fibers; Increased the onset time of the spastic muscle fatigue; Reduced pain intensity	Not mentioned	(29)
Limb spasticity	Equipment: diode laser; Wavelength: 808 nm; Power density: 3.18 W/cm^2^; Energy density: 159.24 J/cm^2^	Biceps brachii muscle with 16 irradiation points	Each irradiation point for 50 s; 3 times a week, for 4 weeks (10 sessions total)	Range of elbow motion; Isometric testing; Surface electromyography (EMG)	Increased range of elbow motion; Increased recruitment of muscle fibers	Not mentioned	(30)
Limb spasticity	Equipment: diode laser; Wavelength: 780 nm; Power density: 3.18 W/cm^2^; Energy density: 127.4 J/cm^2^	Brachial biceps and brachial triceps; 16 irradiation points for each muscle.	Each irradiation point for 40 s; 3 times a week, for 10 sessions total	Visual analogue scale (VAS); Blood lactate level test; Range of elbow motion; Isometric testing; Electromyography (EMG)	Increased range of elbow motion; Reduced pain intensity; Increased peak torque; Decreased blood lactate level;	Not mentioned	(31)
Limb spasticity	①Equipment: super pulsed infrared laser; Wavelength: 905 (± 1) nm; Power density: 0.71 mW/cm^2^; Energy density: 0.054, 0.162, and 0.271 J/cm^2^; ②Equipment: red LED; Wavelength: 640 (± 10) nm; Power density: 16.67 mW/cm^2^; Energy density: 1.27, 3.8, and 6.35 J/cm^2^;③Equipment: infrared LED; Wavelength: 875 (± 10) nm; Power density: 19.44 mW/cm^2^; Energy density: 1.48, 4.43, or 7.41 J/cm^2^	Knee extensor muscle with 9 irradiation points, knee flexor muscle with 6 irradiation points, and plantar flexor muscle with 2 irradiation points	Four times PBMT/sMF with total energies delivered per site (0 J, 10 J, 30 J, and 50 J) within 4 weeks.	6 min walk test (6MWT); Timed Up and Go (TUG) assessment	Improved functional mobility	No harmful thermal effect	(32)
Hemiplegic shoulder pain	Equipment: laser; Wavelength: 904 nm; Power density: 30 mW/cm^2^; Energy density: 3 J/cm^2^	Glenohumeral joint with 9 irradiation points	Each irradiation point for 30 s; 3 times/week, for 4 weeks	Fugl-Meyer Assessment; visual analog scale (VAS); Shoulder Pain and Disability Index (SPADI); Barthel index; Brunnstrom Recovery Stage(BRS); Modified Ashworth Scale (MAS)	Reduced pain intensity; Improved upper extremity functions and disability	Not mentioned	(38)

## Transcranial PBM

The tPBM is the major paradigm for treating neurological disorders. For post-stroke dysfunctions, tPBM has been shown to improve cognitive function and aphasia.

## Cognitive impairment

Post-stroke cognitive impairment is primarily characterized by poor executive function, memory, attention, language, and visuospatial function, and it often occurs within 6 months of a stroke. Approximately 1/3 of stroke patients experience cognitive impairment, which is associated with poorer functional outcomes, increased dependency, higher mortality, and an elevated risk of recurrent stroke. Previous studies have reported that tPBM can enhance cognitive function in healthy individuals and has therapeutic potential for cognitive impairment in conditions like mild cognitive impairment (MCI), TBI, and AD (19, 20). Recent studies have begun to explore the application of PBM for cognitive impairment in stroke patients, with promising preliminary results. Paolillo et al. supplemented neuromuscular electrical stimulation (NMES) therapy in post-stroke hemiplegic patients with tPBM. After 3 months of combined NMES and PBM treatment, stroke patients exhibited significant cognitive improvement, whereas the patients receiving NMES alone showed no significant cognitive gains. In their study, thermographic imaging of the parietal and frontal regions before and after the laser intervention revealed a marked subsequent increase in cutaneous temperature in each region of interest, implying that enhanced blood flow could improve local oxygenation and nutrient delivery. The researchers suggested that the hemodynamic, photochemical, and photophysical effects of PBM may promote neuroplasticity, thereby supporting functional and cognitive recovery, especially when combined with NMES (21). In another randomized trial, Huang et al. investigated the effect of red-light PBM on cognitive function in stroke patients with cognitive impairment. A total of 90 stroke patients were enrolled and randomly assigned in a 1:1 ratio to either the red light therapy group or the sham red light therapy group. The patients in the red light therapy group wore LED helmets and LED belly bands to receive red-light PBM for 30 min, five times per week, while another group of patients wore red light devices that were applied in the same manner but kept switched off (sham stimulation). After a total intervention duration of 3 months, two groups were followed until the six-month study endpoint. At the 6-month follow-up, patients treated with PBM exhibited significantly higher Montreal Cognitive Assessment (MoCA) and Mini-Mental State Examination (MMSE) scores than before treatment and those in the sham group, indicating that red-light PBM improved post-stroke cognitive impairment. In addition, the PBM group exhibited a lower rate of stroke recurrence during follow-up. The researchers also analyzed differences in key enzymes and metabolites involved in formaldehyde (FA) metabolism in blood and urine samples between the groups. They suggested that FA contributes to cognitive decline, and 630 nm red light can degrade FA by activating FA-dehydrogenase (FDH) and downregulating semicarbazide-sensitive amine oxidase (SSAO), thereby improving cognitive function. Notably, FDH is widely expressed in the liver, and previous studies have shown that 630 nm red light can penetrate the murine abdominal region to modulate liver function and enhance FDH activity. Accordingly, applying an abdominal light source near the liver can enhance the effect of tBPM on cognitive function in Alzheimer’s model mice (22, 23). Therefore, LED belly bands were used in this randomized trial and offer potential benefits in activating FDN in stroke patients with cognitive impairment (24).

## Aphasia

Aphasia, a common complication of stroke, results from damage to the brain’s language-critical regions and networks. Speech-language therapy (SLT) is an important and effective treatment for post-stroke aphasia (25). However, the therapeutic effect of SLT in clinical practice remains modest. In 2020, some scholars explored the potential benefits of BPM for post-stroke aphasia. Naeser et al. reported that red/near-infrared tPBM treatment improves naming ability in persons with aphasia (PWA) due to left hemisphere (LH) stroke. They recruited 6 stroke patients with aphasia, comprising 3 cases with mild–moderate nonfluent Broca’s aphasia, 2 cases with recovered nonfluent Broca’s aphasia, and 1 fluent case with a rare unilateral word deafness, and designed four protocols. Protocol A: LED cluster heads were placed on the bilateral hemispheres and supplementary motor areas (SMAs) at the vertex. Protocol B: LED cluster heads were placed on the ipsilesional LH side. Protocol C: LED cluster heads were placed on the ipsilesional LH side and one midline placement over the mesial prefrontal cortex (mPFC) at the front hairline. Protocol D: LED cluster heads were placed on the ipsilesional LH side and over two midline nodes of mPFC and precuneus (high parietal). Each protocol requires the patient to receive 18 sessions of tPBM treatment (3 times per week with at least 48 h between treatments). Patient 1 and Patient 2 first received Protocol A, and the results of the Philadelphia Naming Test (PNT) indicated that their picture naming was not improved. In contrast, their naming ability was significantly improved after Protocol B. Following Protocol A, the overt picture-naming task fMRI scans of Patient 1 revealed increased activation in bilateral hemispheres, including the right frontal, temporal, and parietal regions, contra-lesional cortical areas, and bilateral SMAs at the midline vertex. The distinct LED placements in Protocols A and B each influenced different surface brain cortex areas, yet only the ipsilesional left-hemisphere placement correlated with a significant improvement in naming performance, whereas bilateral placements did not. Consequently, the remaining 4 patients did not receive bilateral LED placements for subsequent research. Patient 5 and Patient 6 responded well to the Category naming test or Picture naming test after Protocol D. In contrast, Patient 3 and Patient 4 did not exhibit a good response in Category naming after Protocol C. The researchers suggested that Protocol D provides significant improvement in naming ability for patients with aphasia, indicating that tPBM can serve as a noninvasive treatment for post-stroke aphasia (26).

## Peripheral PBM

Peripheral PBM directly irradiates the hemiplegic limbs. Some scholars have termed this paradigm, which targets peripheral tissues or organs, as “remote PBM” (27). Direct PBM irradiation of a hemiplegic limb reduces spasticity and pain in post-stroke patients.

## Limb spasticity

Spasticity is a neuromuscular dysfunction characterized by increased muscle tone. Post-stroke limb spasticity reduces physical activity and induces compensatory motor patterns, leading to paretic muscle atrophy and weakness, which strongly correlates with reduced motor function. Since PBM exhibits therapeutic effects on muscular disorders and can prevent muscle fatigue, dos Reis et al. investigated the immediate effects of low-intensity laser (808 nm) on fatigue and strength in the spastic muscles of spastic hemiparetic patients. The laser probe was held stationary in skin contact at 90° with slight pressure and applied to 30 points of each rectus femoris and vastus muscles. Following PBM, the patients received isometric testing and a blood lactate level test. Compared with the condition without PBM, patients’ peak torque was significantly increased, and the concentration of lactate in the spastic post-exercise muscle was significantly decreased, indicating that PBM improved muscle performance without directly increasing fatigue, thereby representing an effective treatment for spastic muscles. The researchers suggested that PBM activates mitochondrial function in muscle tissue to elevate intracellular ATP, ensuring greater energy availability for cellular activities. In addition, PBM induces smooth muscle relaxation and increases peripheral microcirculation via the temporary release of NO, leading to improved reperfusion blood and decreased lactic acid concentration and muscle fatigue (28). In a subsequent experiment, dos Reis et al. investigated the effect of PBM on lower limb spastic muscle activity in chronic stroke patients. The laser probe was sited on the rectus femoris and vastus medialis from the paretic limb and irradiated 30 points per muscle in sequence. Following PBM, the patients were evaluated using the visual analogue scale (VAS), an isokinetic dynamometer, and an electromyography (EMG). The researchers observed a significant reduction in VAS pain scores, a delayed onset of muscle fatigue, and an increased torque peak in patients, indicating that PBM may enhance muscle fiber recruitment and reduce pain intensity and muscle fatigue in stroke patients with spasticity. They suggested that PBM can modulate the myoelectric activity of spastic muscles, promoting recruitment of more motor units in the spastic rectus femoris, and exerts analgesic effects through muscle relaxation and stimulation of endogenous opioid release (29). Afterwards, these researchers investigated the effects of PBM on the spastic muscle behavior in chronic post-stroke patients. A total of 15 chronic hemiparetic patients with biceps spasticity received 10 sessions of PBM treatment at the biceps brachii. The results from the range of motion (ROM) test of elbow, electromyography (EMG), and dynamometry revealed that PBM significantly increases the ROM of the elbow, average torque, and EMG root mean square (RMS), indicating that PBM improves ROM of the spastic elbows, muscle activation, and fiber recruitment of the spastic biceps muscle in chronic stroke patients. Moreover, the researchers found that PBM intervention before exoskeleton-assisted functional treatment yielded greater improvement of performance during exercises, resulting in increased ROM and recruitment of muscle fibers. They suggested that PBM associated with exoskeleton-assisted functional treatment may offer a new alternative for treating post- stroke neuromuscular sequelae (30). In a recent study, dos Reis et al. investigated the effect of PBM on the relief of pain in the paretic upper limb in post-stroke patients with spastic hemiparesis. They recruited 15 post-stroke individuals and measured the pain and ROM of the paretic upper limb before and after 10 sessions of PBM treatment. VAS results showed a 38% reduction in pain reported by hemiparetic patients following PBM treatment. Notably, elbow extension in the paretic limb increased by 46.1% compared to pre-treatment levels. This study indicated that PBM significantly reduces pain levels and improves the ROM in upper limb spasticity (31). Together, the series of clinical trials mentioned above demonstrated that PBM irradiation on hemiplegic limbs can significantly improve motor impairments in post-stroke patients with spastic hemiplegia by reducing muscle fatigue and pain levels, and expanding joint range of motion.

In another study, Casalechi et al. reported that PBM combined with a static magnetic field (sMF) had acute positive effects on functional mobility in stroke survivors by reducing muscle weakness in both lower limbs. They recruited 12 stroke patients with hemiparesis who could walk barefoot, with or without a gait-assistance device (cane), and complete the 6-min walk test (6MWT) and Timed Up and Go (TUG) assessment. A cluster of 12 diode devices, which can generate a magnetic field of 35 mT, was placed at 9 sites on the knee extensors, 6 sites on the knee flexors, and 2 sites on the plantar flexors of both lower limbs. Finally, 10 patients completed the 4-week treatment. During the treatment period, patients showed significant improvements in both 6MWT and TUG tests after receiving PBM (30 J per site) combined with sMF treatment. The researchers concluded that this combined approach benefits functional mobility after stroke, representing a promising alternative for treating motor impairments during rehabilitation (32).

## Hemiplegic shoulder pain

Hemiplegic shoulder pain (HSP), a common complication after stroke, often reduces the range of motion and limits motor function of the shoulder. The etiology of HSP is multifactorial, encompassing shoulder subluxation, muscle spasticity/flaccidity, adhesive capsulitis, rotator cuff injury, and shoulder-hand syndrome (33, 34). A recent meta-analysis indicates that approximately 22–47% of patients develop this shoulder pain (35). PBM therapy has demonstrated efficacy in alleviating pain and improving function in various shoulder and neck pathologies (36). As early as 2009, Karabegović et al. used an 830 nm laser to irradiate pain points in the shoulders and area of swelling on the dorsum of the hand, alleviating shoulder pain and shoulder-hand syndrome (33). Subsequently, Jan et al. suggested that PBM was superior to interferential current therapy for reducing pain and enhancing the satisfaction in stroke patients with shoulder pain (37). In a recent study, researchers compared the efficacy of PBM and neuromuscular electrical nerve stimulation (NMES) in the treatment of HSP. In this trial, one group of 25 patients received 904 nm laser irradiation at nine shoulder joint points three times weekly for 4 weeks, while another group of 25 patients underwent NMES at motor points near the supraspinatus and deltoid muscles five times weekly for 4 weeks. Researchers have found that administering PBM or NMES alongside classical physical therapy exercises (passive, passive-assisted, active ROM, stretching, and strengthening exercises) can effectively alleviate shoulder pain and improve shoulder function. Despite their distinct mechanisms of action, PBM and NMES produced no statistically significant differences in pain relief or functional improvement (38). In short, PBM can ameliorate HSP, leading to improved upper extremity function and reduced disability in stroke patients.

## Challenges of PBM for post-stroke dysfunctions

The above clinical studies confirm that PBM can ameliorate post-stroke dysfunctions by directly irradiating the skull or muscle tissue. However, the diversity of devices and treatment protocols across these trials complicates the reproducibility of PBM’s therapeutic benefits and underscores inherent translational challenges.

## Treatment parameters

In previous reviews on PBM treatment, scholars have consistently expressed concern about the treatment parameters (1, 2, 4, 39). The treatment parameters of PBM, including wavelength, power density, placement location, and treatment duration, are recognized as critical determinants of PBM efficacy. (1) Wavelength can influence both light absorption and penetration depth. It is widely believed that near-infrared wavelengths have superior tissue penetration. Lapchak et al. reported that the transmittance of 810 nm light in mouse skulls was 40% but only 4.2% in human skulls (40). NIR II (1000-1700 nm) light exhibits greater penetration than NIR I (630-900 nm) light and appears more promising. For instance, 1,064 nm laser irradiation effectively improves brain function due to its high skull penetration and favorable safety profile (41, 42). In addition, NIR light also varies by the irradiated anatomical region of the skull. Jagdeo et al. found that the penetration of 830 nm light differed across the temporal, frontal, and occipital regions of human cadaver heads. Percent penetrance of light through sagittal sections of the occipital region was 11.7%, which was better than 0.9% at the temporal region and 2.1% at the frontal region (43). (2) Regarding power density, reports said that PBM’s energy densities at 10–84 J/cm^2^ are effective in humans. Notably, there is a biphasic dose–response relationship consistent with the Arndt-Schulz Law during PBM treatment. Low doses produce a stimulatory effect, whereas high doses may not confer additional benefits and can even induce a negative response (5). (3) Although treatment duration and repetition regimen are also important, few studies have specifically examined how irradiation time affects PBM or compared the efficacy of different treatment protocols. Salehpour et al. suggested that it is unreasonable to expect a single session of PBM in stroke patients to have the best effects, and repeated PBM is required (4). However, the number of PBM treatments still needs further clinical trial evidence to achieve consensus.

## Lighting equipment

Equipment is another crucial aspect for PBM. The light source for PBM is usually a laser or light-emitting diode (LED) (44). A laser source can produce a single wavelength of light, with high tissue penetration and a constant beam width. The limited spot size of lasers may be insufficient for large areas, requiring repeated single-beam irradiation for large organs. In contrast, LEDs offer light across a range of wavelengths, with high energy efficiency and low heat generation. LED arrays can be mounted on ergonomic arrays to deliver energy efficiently over broader surfaces, such as the brain. LED devices can be designed for various shapes and sizes, such as LED helmets, girdles, and glasses, which facilitates their use in different treatment areas. However, the light-emitting devices used in different clinical studies vary in key parameters, such as beam profile, spot size, pulse mode, delivered energy, and operator technique. The absence of uniform equipment design standards and medical device safety guidelines further restricts the clinical translation of PBM.

## Light delivery methods

Beyond transcranial delivery, light irradiation through the intracranial, nasal, and/or oral cavity represents an alternative route for stroke, which has been used to improve symptoms of dementia and PD (4). Intracranial delivery of light via an implanted optical fiber can deliver the light to subcortical regions. However, as an invasive operation, it may cause tissue incompatibility and inflammatory reactions in the brain. Intranasal light delivery can directly irradiate the subcortical structures (e.g., hypothalamus, thalamus, amygdala, hippocampus) and the orbitofrontal cortex. Oral light delivery can irradiate the pons and cerebellum. Different light delivery methods can be used to achieve precise intervention based on the different lesions in the brain of the stroke patient. Existing evidence suggests that the depth of action of PBM may apply to superficial muscle tissue (45). Researchers often choose to cover target muscle tissue by distributing multiple irradiation points or expanding the irradiation area. However, this treatment regimen frequently neglects muscle physiology, including origin and insertion points, and contraction patterns. In addition, the role of antagonistic muscles in patients with hemiplegic limbs also needs attention.

## Discrepancy between preclinical and clinical findings

The majority of preclinical studies exhibited encouraging findings. The tPBM therapy has shown promising results in reducing infarct size, promoting neurological function, improving motor function, and enhancing cognitive and learning abilities in animal models (46–50) (Table 2). These studies have primarily involved rodent models. Even though tPBM can reach deeper brain regions of rodents through the skull, such as the dorsal hippocampus and amygdala, achieving similar depth in humans is difficult without intracranial or oral light delivery. Variations in light parameters/treatment dosage and the devices utilized in many animal studies lead to discrepancies in biological effects, even when employing the same light wavelength. Therefore, extrapolating the results of animal experiments to clinical application in humans requires caution. The discrepancy between preclinical and clinical findings poses a challenge in clinical translation of PBM.

**Table 2:** Literature examples of photobiomodulation on stroke in animal models in recent 5-years.

Animal model	Irradiation sites	Parameters of light	Duration time	Evaluation tools	Outcomes	References
SD rats	The area of the scalp underlying the infarct injury	Equipment: Lasers; Wavelength: 808 nm; Power density: 350 mW/cm^2^	2 min/day for 7 days	Fluorescence staining and imaging	Alleviated dendritic and synaptic injury in the peri-infarct area of the cerebral cortex; Inhibited neurotoxic astrocytic polarization	(46)
C57BL/6 J mice (Wild-Type and eNOS knockout)	Top of the skull	Equipment: Lasers; Wavelength: 1064 nm; Power density: 50 mW/cm^2^	Pre-treatment: 4 days before stroke; 5 min/day for 4 days	Cerebral blood flow (CBF) measurement; Evaluation of neurological deficit, infarct volume, and eNOS phosphorylation;	Reduced infarct volume; Increased eurologic score; Increased eNOS phosphorylation	(47)
SD rats	The area of the scalp underlying the infarct injury	Equipment: Lasers; Wavelength: 808 nm; Power density: 350 mW/cm^2^; Energy density: 42 J/cm^2^	2 min/day for7 days	Behavioral assessments; Morphometric measurement of the brain vasculature; Testosterone concentration measurement	Alleviated behavioral deficits; Improved vascular morphology; Increased the cerebrovascular testosterone concentration	(48)
SD rats	Cranial window surface	Equipment: Lasers; Wavelength: 755 nm; Power density: 20 mW/cm^2^	10 min/day for 7 days	Cognitive behavioral testing; MRI; pro-inflammatory cytokines quantification	Decreased infarct volume; Decreased pro-inflammatory cytokine levels; Improved neurological cognitive function	(49)
C57BL/6 J mice	Sensorimotor cortex	Equipment: implantable LEDs; Wavelength: 630, 850, and 940 nm; Power density: 17 mW/cm^2^	Protocol 1: 10 min each time, twice a day, starting from 3 days before ischemic brain injury to determine the preventive effect; Protocol 2: 10 min each time, twice a day, starting from 4 h after the ischemic insult for 3 days to assess the therapeutic effect; Protocol 3: 10 min a day for 7 days to assess the therapeutic effect on chronic cognitive impairment	Evaluation of infarct volume and Neurological Score; Vestibular motor function testing; Cognitive behavioral testing; Immunofluorescence Staining	630 nm PBM reduced infarction volume and neurological impairment after ischemic stroke, improved the capability of spatial learning and memory in the chronic poststroke phase, and attenuated AIM2 inflammasome activation and inflammasome-mediated pyroptosis.	(50)

## Summary and outlook

**Figure 2:**
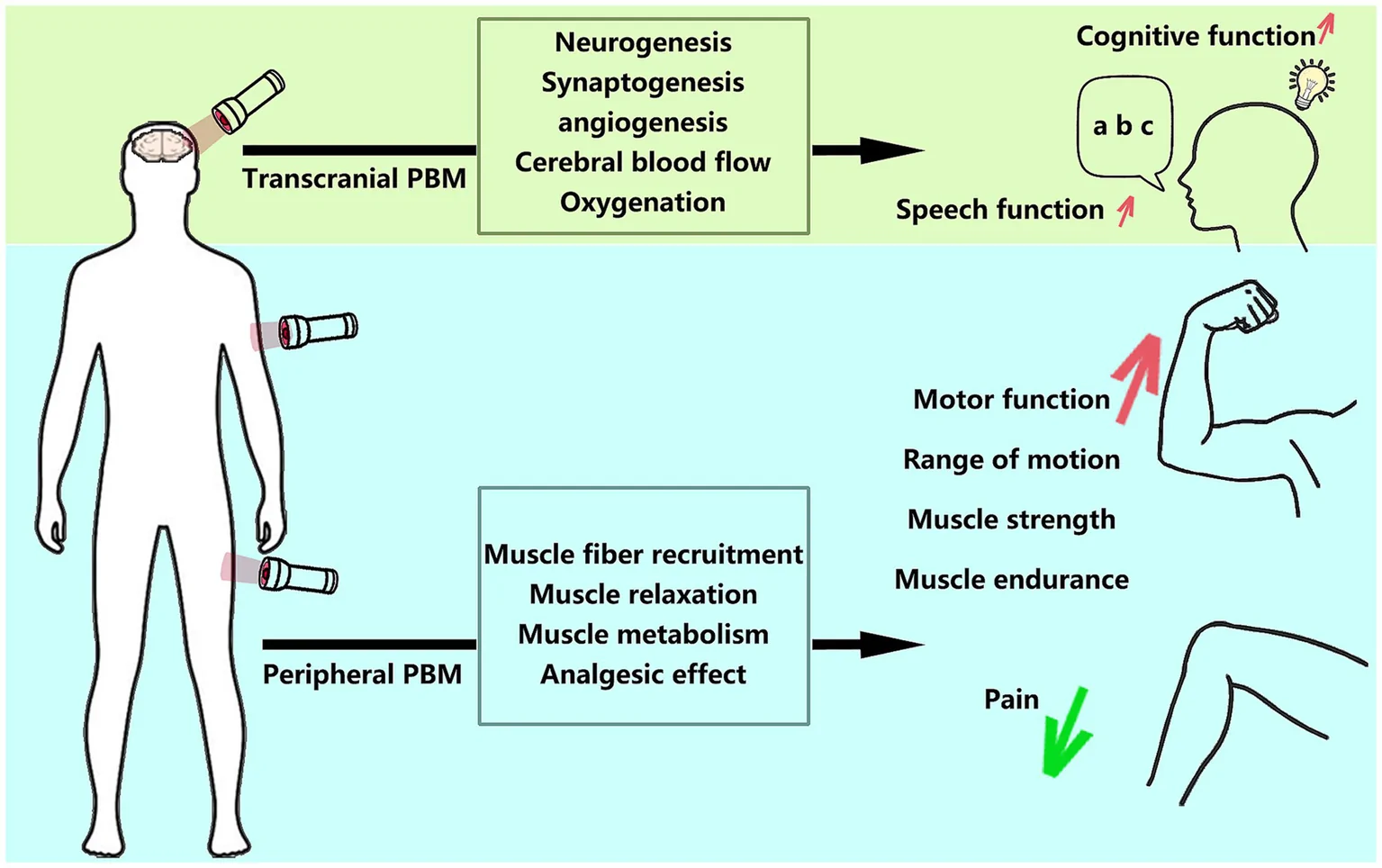
Schematic representation of PBM ameliorates post-stroke dysfunctions.

In 2025, an international multidisciplinary team of more than 20 experts published an evidence-based clinical practice guideline for PBM. This guideline establishes PBM as an effective treatment for peripheral neuropathy, androgenic alopecia, wound ulcers from multiple etiologies, decubitus ulcers, pain from diabetic foot ulcers, and acute radiation dermatitis (51). By contrast, clinical research on PBM for stroke remains limited, and a formal expert consensus is still absent, which may require more reliable clinical evidence. Therefore, this review summarizes the recent clinical application of PBM for post-stroke dysfunctions. We can learn from it that at present, beyond cranial applications for cognitive impairment and aphasia, PBM also yields positive outcomes for limb spasticity and pain when applied directly to paretic limbs (Figure 2). Theoretically, cranial PBM enhances mitochondrial function and creates a favorable microenvironment for remodeling of synaptogenesis, neurogenesis, and neuroplasticity. Peripheral limb application induces smooth muscle relaxation and improves the peripheral microcirculation and muscle metabolism.

Of course, we must acknowledge that the efficacy of PBM in regulating brain function remains challenged by certain research findings, owing to its inherent limitations. A recent study investigated whether the NIR light emitted by a 905 nm super-pulsed laser (peak power: 300 W) and two LED helmets operating at 810 and 1,070 nm could induce protective mitochondrial stress responses in SH-SY5Y neuroblastoma cells and *Caenorhabditis elegans* (*C. elegans*) by stimulating COX activity after penetrating a human skull. They concluded that the NIR light dose delivered by the tested devices after penetrating the human skull was insufficient to stimulate mitochondrial activity (52), indicating that tPBM must overcome the challenge posed by skull thickness. Notably, some new attempts provide the possibility of overcoming the inherent limitations of tPBM. An experimental attempt by Yeh et al. using intravascular laser irradiation of blood (ILIB) to improve stroke risk biomarkers and neurological function may suggest new directions for PBM treatment sites and light sources for stroke. This study indicated that red-light (650 nm) and blue-light ILIB (415 nm) both improved the motor and balance performance of post-stroke patients, with red-light ILIB further enhancing upper-limb function in older adults and cognition in younger participants (53). Moreover, the combination of nanomaterials and tPBM will help us overcome the challenge of transmitting light to deeper structures in the brain. For example, Upconverting Nanoparticles can absorb low-energy photons in the “third optical window” (1550–1870 nm), which are optimal for light penetration into brain tissue, and then transfer the energy to monochromatic emission in the visible or NIR light. When successfully delivered to the brain, UCNPs can induce optically manipulated neuronal activity after special light irradiation, allowing more efficient PBM of the brain (54).

In contrast to tPBM, peripheral limb application may be more suitable as an adjunctive therapy in the rehabilitation of post-stroke dysfunctions. First, by alleviating spasticity, improving range of motion, and reducing pain and muscle fatigue in hemiplegic limbs, peripheral PBM enables patients to take a more active role in physical therapy (PT), occupational therapy (OT), and exoskeleton-assisted functional training. Second, combining peripheral PBM with other noninvasive brain stimulation (NIBS) techniques, such as transcranial magnetic stimulation (TMS) and transcranial electrical stimulation (TES), may further alleviate motor impairment. Third, peripheral PBM may allow a reduction in the dosage of antispastic medications like baclofen or tizanidine, thereby minimizing adverse reactions and enhancing the safety of pharmacotherapy. These potential advantages of peripheral PBM also offer new ideas for developing future combination treatment strategies targeting post-stroke dysfunctions.

In summary, although an increasing number of clinical studies have demonstrated the benefits of PBM for post-stroke dysfunctions, the field remains in its early stages. PBM currently faces many challenges in stroke research and future clinical translation, requiring not only more supporting clinical evidence but also further research on its mechanism of action.
